# Effects of different irrigation levels on European corn borer (*Ostrinia nubilalis (Hübner)*) populations

**DOI:** 10.1371/journal.pone.0212595

**Published:** 2019-03-19

**Authors:** M. Cüneyt Bağdatlı

**Affiliations:** Nevsehir Hacı Bektaş Veli University, Engineering and Architecture Faculty, Department of Biosystem Engineering, Nevsehir/Turkey; Nigde Omer Halisdemir University, TURKEY

## Abstract

In In this study, the effects of different irrigation treatments (I_100_, I_50,_ I_30_) on the European corn borer (*Ostrinia nubilalis hübner)* populations in different corn genotypes (Ada-9510, Samada-07, Ada-523, Kompozit Arifiye) were investigated. As the average, the highest yield (1582.33 kg da^-1^), low number of live larva + pupa per plant (2.70), tunnel length (7.73 cm) were observed in I_100_ x ADA-523 interaction while the lowest yield (896.00 kg da^-1^) was observed in I_30_ x Kompozit Arifiye interaction. The largest European corn borer population was observed in full irrigation (I_100_) and the lowest population was observed in excessive water deficit (I_30_) treatment. The European corn borer preferred the Kompozit Arifiye genotype. It was concluded that the genotypes should be tested under water-stressed and non-water-stressed conditions for resistance to the European corn borer and live larva and tunnel length could be used as a screening parameter.

## Introduction

In the world, there are several pests with negative impacts on corn culture in Turkey such as European corn borer (*Ostrinia nubilalis hübner*) and Mediterranean corn borer (*Sesamia nonagrioides lefebvre*) which are among all the most important ones. While the European corn borer is widespread in Europe, America and Turkey, the Mediterranean corn borer is widespread in in Mediterranean countries such as Spain, France, Italy, Greece and Turkey which is why it is called “Mediterranean Corn borer” in literature [1–3]. In the Çukurova district of Turkey, the European corn borer reproduces 3 to 4 times and the Mediterranean corn borer reproduces 4 to 5 times a year [4]. It has been reported in previous studies that the Mediterranean corn borer represented 38.95% of the corn pests in Çukurova while the European corn borer constituted 61.15%. It was indicated that these ratios were 70% for Mediterranean corn borer and 30% for European corn borer based on infected number of plants and were 87% for Mediterranean corn borer and 13% for European corn borer based on live larva + pupa ratio per plant [5]. The Mediterranean corn borer and the European corn borer create damages in all the organs of the corn plants, except for the roots. During the harvest, approximately 95% infections were observed in untreated fields [6] and approximately 30% yield loss was experienced [7]. Researchers reported that the Mediterranean corn borer resulted in between 0 and 10% yield losses in the first crop and 80 and 100% yield losses in the second crop of corn. [8].

A study was carried out in Nebraska with irrigated and non-irrigated corn plots in which the European corn borer population was reported to be larger in irrigated plots than in non-irrigated plots [9]. In another study, the effects of different irrigation treatments (I_100_, I_80_, I_60_, I_40_, I_20_, I_0_) on the Mediterranean corn borer and the European corn borer populations were investigated and a larger population (27.167 per plant) of both species was reported in full irrigation treatments (I_100_) than in non-irrigated treatments (I_0_) (13.50 per plant) [10;11].

Stressors stimulate the formation of reactive oxygen species in plants. These reactive species are usually called radicals and attack various surfaces in plant tissues and create damages. Free radicals mostly attack polyunsaturated fatty acids in cell membranes. The interaction of unsaturated fatty acids with these radicals alters the saturated and unsaturated fatty acid ratios of cell membranes [12]. Researchers reported increased total saturated fatty acid ratios with abiotic stressors [13].

Eicosapolyenoic acids (EP), which do not occur in taller plants, including an oxidative burst and the transcriptional activation of genes involved in phytoalexin synthesis, lignification, programmed cell death, and other responses typically associated with the hypersensitive response (HR) to pathogens [14]. Previous studies have reported that common fatty acids in plant pathogenic oomycetes, and signals for immune responses and central nervous system development in mammals function as conserved signaling molecules across eukaryotic kingdoms. The fatty acids released during the infection of plants may serve as novel pathogen-associated molecular patterns (PAMPs) that engage plant signaling networks to induce resistance to pathogens [13]. The levels of free fatty acids increase in response to various stresses and play a pivotal role in plant–microbe interactions. For example, fatty acid synthesis in the obligate biotrophism of arbuscular-mycorrhizal fungi is dependent on plant-derived C16 FAs [15]. Furthermore, eggplants with enhanced levels of palmitoleic acid (16:1) exhibited increased resistance to *Verticillium dahliae*, suggesting an increase in the production of plant 16:1 as a viable approach to enhance crop resistance to fungal diseases [16].

Several studies were performed to determine the quantitative sections in corn plants which developed resistance to the European corn borer [17, 18, 19, 20, 21, 22, 23]. However, there are no studies in the literature regarding the effects of different irrigation levels (I_100_, I_50_, I_30_) on the European corn borer population in corn plants. Therefore, sufficient literature about the present research topic could not be provided.

This study was conducted to investigate the effects of different irrigation levels (I_100_, I_50,_ I_30_) on tunnel length and European corn borer population in different corn genotypes (Ada-9510, Samada-07, Ada-523, Kompozit Arifiye).

## Material and methods

**Fig 1:**
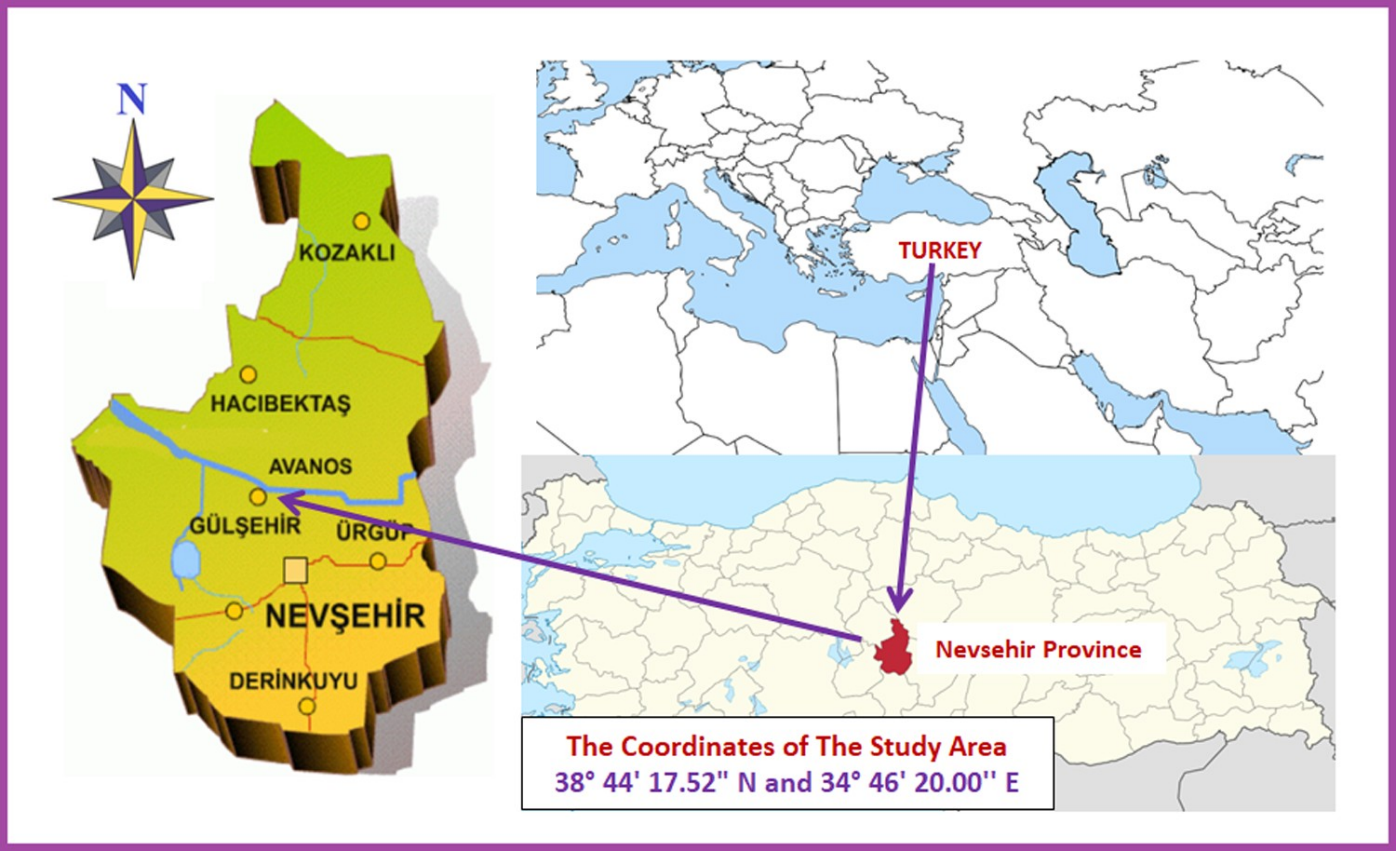
Location of the study area.

Experiments were carried out on the experimental fields located in the province of Nevşehir, Turkey during the maize growing seasons of 2015. The research site has an altitude of 1045 m and is located on 38° 44' 17.52" N and 34° 46' 20.00'' E. (Fig 1)

In this study, The Ada-9510, Samada-07, Ada-523, Kompozit Arifiye corn genotypes were used as the plant material. The long-term and annual climate data of the research site are provided in Table 1.

**Table 1:** Some climate datas of the study areas (2015 year).

Months 2015	Min. Temp. (°C)	Max. Temp. (°C)	Aver. Temp. (°C)	Total Rain (mm)	Humudity (%)
January	-1.3	9.7	0	51.6	71.3
February	-4.5	11.1	1.6	31.6	63.5
March	2.2	14.8	6.3	71.6	62.0
April	3.8	17.4	11.8	20.6	53.2
May	10.6	20.4	17.1	41.5	48.6
June	14.5	28.7	20.7	41.6	60.7
July	20.3	30.6	25.9	8.4	42.2

According to the annual climate data of the research site, the total precipitation of Nevşehir is 421 mm. Precipitation is 41.6 mm in June, 8.4 mm in July. The average temperature is 0.8°C in winter, 10°C in spring, 20.9°C in summer and 11.7°C in autumn.

Soil samples were taken before the sowing process from 0–90 cm soil profile (from three depth segments as 0–30, 30–60 and 60–90 cm). Soil moisture content at field capacity (33 kPa), bulk density, organic matter, texture and permanent wilting point analyses, water holding capacity at permanent wilting point were determined [24, 25]. The soil physico-chemical characteristics are provided in Table 2.

**Table 2:** Some soil physical and chemical soil characteristics of the research site.

Soil Depth (cm)	Sand (%)	Clay (%)	Silt (%)	Organic material (%)	Texture Class	Bulk Density (g/cm^3^)	Field Capacity (%)	Wilting Point (%)
0–30	34	22	44	1.04	L	1.47	20.99	8.38
30–60	34	28	38	0.46	CL	1.58	21.55	9.37
60–90	32	24	44	0.21	L	1.48	22.74	10.01

C: Clay, CL: Clay Loam, L: Loam

The experimental soil had low electrical conductivity and salinity, low phosphor content, high potassium content and medium organic matter content. Lime levels did not cause any problems for plant growth. In Nevşehir, the field capacity was 22.74%, permanent wilting point was 10.01% and soil bulk density was 1.48 gr cm^-3^. Irrigation water quality parameters were determined in accordance with the method specified [26]. Irrigation water quality class was C_4_S_2_ with an average EC value of 0.27 dS m^-1^ and a pH value of 7.53 (Table 3).

**Table 3:** The quality characteristics of Irrigation water in study areas.

pH	EC (μs/cm)	Ca (me/L)	Mg (me/L)	Na (me/L)	K (me/L)	CO_3_ (me/L)	HCO_3_ (me/L)	Cl (me/L)	SO_4_ (me/L)	SAR	Class
7.49	2719	8.9	3.0	15.5	0.23	0.00	14.62	3.22	9.56	6.43	C_4_S_2_

**Fig 2:**
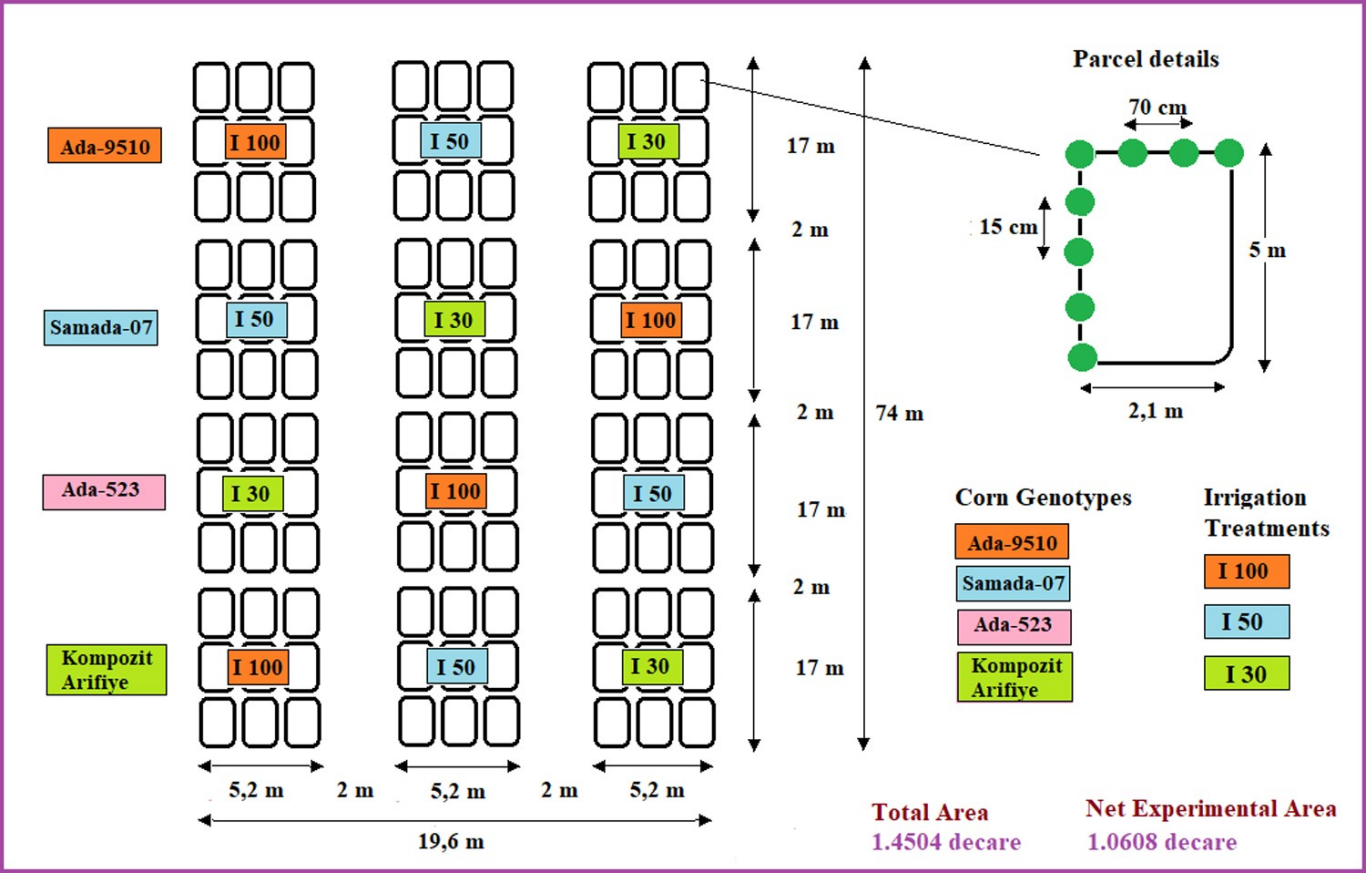
Experimental design of the study area.

Experiments were conducted using randomized blocks-split plots experimental design with 3 replications with genotypes (Ada-9510, Samada-07, Ada-523, Kompozit Arifiye) on main plots and irrigation treatments (I_100_, I_50_ and I_30_) on sub-plots (Fig 2).

The irrigation program was scheduled so that irrigation was performed once a week. Treatments were selected as full irrigation (I_100_) in which 100% of depleted moisture was supplied, water-stressed (WS) treatment (I_50_) in which 50% of depleted moisture was supplied and excessive water stress treatment (I_30_) in which 30% of depleted water was supplied. Therefore, one full irrigation treatment and two deficit irrigation treatments were created.

Drip irrigation was used to perform the irrigations. A lateral line (20 mm and 4 atm operational pressure, 0.33 m apart 4 L h^-1^ drippers) was placed along each plant row. Soil infiltration rate was measured as 8 mm h^-1^. Deep percolation and surface runoff were not considered. Each plot had a size of 5.2 x 17 m (88.4 m^2^) consisting of 4 plant rows with 70 cm row spacing and 25 cm on-row plant spacing. A buffer zone of 2 m was placed between the experimental plots to prevent interactions.

All of the phosphorus fertilizer (pure 90 kg da^-1^ P_2_O_5_) and one third of nitrogen (28 kg da^-1^ N) were supplied during the sowing process. The rest of the nitrogen was given when the plants were 40–50 cm tall.

The gravimetric moisture content of each layer (0–30, 30–60 and 60–90 cm.) was converted into depth using Eq 1.

$$\text{d}\;=\;\left({\text{Pw-Pw}}_{\text{AW}}\right)\;\text{x As x D/100}$$ (1)

Where; d is soil moisture content in depth (mm), Pw is field capacity (%), Pw_AW_ is the moisture content of each layer (%), As is soil unit weight (g cm^-3^) and D is layer depth (mm). The volume of the water to be applied was calculated by using Eq 2.

$${\text{d}}_{\text{T 0-90}}{=\text{d}}_{\text{(0-30)}}{\text{+ d}}_{\text{(30-60)}}{\text{+ d}}_{\text{(60-90)}}$$ (2)

Where; d_T 0–90_ is soil moisture at 0–90 cm soil profile (mm), d _(0–30)_ is soil moisture at 0–30 cm soil profile (mm), d _(30–60)_ is soil moisture at 30–60 cm soil profile (mm) and d _(60–90)_ is soil moisture at 60–90 cm soil profile (mm).

The volume of the water to be applied to each plot was calculated by Eq 3.

$${\text{V}=\text{d}}_{\text{T}}{\;\text{x A x U}}_{\text{o}}\;\text{x P}$$ (3)

Where; V is the volume of water to be applied (L), A is plot size (m^2^), U_o_ is deficit ratio (%) and P is cover ratio (%).

The plant canopy width was divided by row spacing to obtain cover ratios (CR). The ratio was taken as 0.30 and 0.80 for the cover ratios of 30% and 80%, respectively. The principles specified by researchers were used to find the amount of water to be used in each plot [27].

Plant water consumptions were calculated by using Eq 4 [28].

$${\text{ET}}_{\text{a}}{=\;\text{P}\;+\;\text{I}\;–\;\text{R}}_{\text{f}}{–\;\text{D}}_{\text{p}}\pm \;\Delta \text{S}$$ (4)

Where; ET_a_ is evapotranspiration (mm), P is precipitation (mm), I is the amount of irrigation water (mm), R_f_ is surface flow (mm), D_p_ is deep percolation (mm) and ΔS is the change in soil moisture (mm).

Harvest was performed when the seed moisture content decreased to 15% to determine the yields. Side rows and 0.5 m space at the top and bottom of the inner two rows were omitted to consider any side effects.

Number of Live Larva + Pupa: In order to determine the effects of different irrigation levels on the number of live larva + pupa; 25 plants were split from the mid-sections into two halves and then the live larva and pupas were counted [5].

Number of Live Larva + Pupa per plant: The total number of larva and pupa was divided by the number of plants to obtain the number of live larva + pupa per plant. The genotypes were then compared with each other based on these numbers.

Number of holes / 100 internodes: To determine the effects of different irrigation levels on the number of holes / 100 internodes ratio, 25 plants from two rows of each genotype were harvested 20 days before the harvest. Leaves were removed from the plants. Then, the number of internodes and number of pest exit holes above and below the corn cobs were counted separately [29, 30].

Number of holes / 100 internodes method:

Below-cob section: Number of holes / 100 internodes = B / A x 100

(Below-cob total number of holes was divided by below-cob total number of internodes and multiplied by 100). B: Below-cob total number of holes, A: Below-cob total number of internodes.

Above-cob section: Number of holes / 100 internodes = E / D x 100

(Above-cob total number of holes was divided by above-cob total number of internodes and multiplied by 100). E: Above-cob total number of holes, D: Above-cob total number of internodes.

Whole plant: Number of holes / 100 internodes = (B + E) / (A + D) x 100

(Below and above-cob total number of holes was divided by below and above-cob total number of internodes and multiplied by 100).

Tunnel (gallery) Length / Plant: To determine the effects of different irrigation levels on tunnel (gallery) length / plant; 25 plants on which number of holes was counted were vertically split into two halves and the lengths of tunnels above and below the cobs were measured with a ruler [29, 30].

Tunnel length / Plant: The genotypes assessed for tunnel length / plant were then assessed for damage index. Plants with tunnel lengths of less than 3 cm were assessed as highly resistant, with tunnel lengths between 3–7 cm were assessed as resistant, with tunnel lengths between 7–10 cm were assessed as moderately resistant, with tunnel length over 10 cm were assessed as sensitive. The tunnels with a length of less than 2.5 cm were not assessed as tunnels. Such holes were considered as entrance-exit of the pests that did not feed from the plant [30].

Each plot had a length of 5.2 m and had 4 rows. Row spacing was 70 cm and on-row plant spacing was 25 cm. Two rows were used for yield and various observations and the other two rows were used for European corn borer damage assessments. European corn borer inoculations were not performed and natural larva was considered in all calculations and assessments. Pesticides were not applied to control main pests (*Ostrinia nubilalis hübner*). In addition, the effects of different irrigation levels (I_100_, I_50_, I_30_) on the Mediterranean corn borer *(Sesamia nonagrioides lefebvre)* populations were not found to be assessable because of the small size of the pest population.

Analysis of variance (ANOVA) was performed in accordance with randomized blocks–split plots experimental design. Significant treatments were then subjected to Least Significant Difference (LSD) multiple comparison tests. Correlation analyses was carried out to identify the relationships between the traits. The directions of the relationships (positive or negative) were determined. The analyses were carried out with JUMP 5.0.1a statistical software [31].

## Results and discussion

Irrigation treatments were initiated on 01 July 2015 when 50% of the available moisture in efficient root zone (0–90 cm) was depleted and the treatments were terminated on 11 September 2015. Harvest was performed on 30 September 2015 in the I_30_ and on 03 October 2015 in the I_100_ treatments (Table 4).

**Table 4:** Changes in yield and physiological properties of corn genotypes depending on the amount of irrigation water.

Treatments	Yield (kg ha ^-1^) ^**^	NOH/100 (number plant^-1^) ^**^	TL/P (cm) ^**^	TNOAL+PP (number plant^-1^) ^**^	Irrigation water (mm)	ETa (mm) **		WUE (kg ha mm ^-1^) **
I_100_ (FI)	1365.33 a	11.61 a	18.468 a	5.75 a	508.25	553.00 a		3.73 a
I_70_ (DI)	1309.58 b	9.89 b	18.465 a	4.54 b	310.75	367.41 b		3.58 b
I_30_ (DI)	1095.66 c	7.48 c	8.99 b	3.38 c	242.00	293.25 c		2.46 c
LSD (0.05)	2.00	0.075	0.33	0.23		6.01		0.042
ADA—523	1430.00 a	10.85 a	9.79 d	3.64 d	510	400.20 b		3.72 a
ADA—9510	1387.00 b	9.62 b	12.63 c	4.38 c	508	408.11 a		3.58 b
SAMADA—07	1252.00 c	9.28 c	17.12 b	4.83 b	509	400.30 b		3.26 c
Kompozit Arifiye	959.00 d	8.89 d	21.67 a	5.38 a	506	410.00 a		2.47 d
LSD (0.05)	8.57	0.095	0.15	0.20		6.99		
I_100_ x ADA-523	1582.33 a	13.70 a	11.66 g	4.54 de	510	547.66 a		2.88 g
I_100_ x ADA-9510	1481.66 b	11.50 b	15.06 e	5.46 c	508	553.33 a		2.67 h
I_100_ x SAMADA-07	1394.00 c	11.10 c	18.26 d	6.19 b	509	558.00 a		2.49 ı
I_100_ x Kompozit Arifiye	1003.33 f	10.16 e	29.36 a	6.82 a	506	553.00 a		1.81 j
I_50_ x ADA-523	1496.33 b	10.61 d	10.48 h	3.70 f	300	361.33 c		4.14 b
I_50_ x ADA-9510	1384.66 c	9.72 f	13.55 f	4.37 e	326	376.66 b		3.68 d
I_50_ x SAMADA-07	1380.66 c	9.62 f	24.41 c	4.70 d	306	349.66 c		3.94 c
I_50_ x Kompozit Arifiye	976.66 g	9.62 f	25.41 b	5.42 c	311	382.00 b		2.56 hı
I_30_ x ADA-523	1211.00 e	8.24 g	7.73 k	2.70 h	243	291.33 d		4.15 b
I_30 X_ ADA-9510	1294.00 d	7.65 h	9.30 ı	3.46 g	246	294.33 d		4.39 a
I_30 X_ SAMADA-07	981.66 g	7.13 ı	8.70 j	3.60 fg	240	293.00 d		3.35 e
I_30_ x Kompozit Arifiye	896.00 h	6.88 j	10.25 h	3.89 f	239	291.33 d		3.04 f
LSD (0.05)	14.85	0.16	0.27	0.35		12.11		1.24

* and **, significant at P≤0.05 and P≤ 0.01 level, respectively; ns, not significant; means in the same column with similar letter are not significantly different from each other; NOH/100, Number of holes /100 internodes; TL/P, Tunnel length/plant; TNOAL+PP, The number of alive larva+pupa in the plant; ETa, Plant water consumptions; WUE, Water use efficiency

With regard to kernel yield, main plot (genotype) x sub-plot (irrigation) interactions were found to be significant (P<0.01). The kernel yields of the first crop varied between 896–1582.33 kg ha^-1^ with the greatest yield from I_100_ x ADA-523 interaction (1582.33 kg ha^-1^) and the lowest yield from I_30_ x Kompozit Arifiye interaction (896 kg ha^-1^). While I_100_ x ADA—523 interaction was placed in Group A, I_30_ x Kompozit Arifiye interaction was placed in Group H. According to the variance analyses for the yields, the irrigation treatments and genotypes were also found to be significant (P<0.01). The greatest yield (1365.33 kg ha^-1^) was obtained from the I_100_ treatment and the lowest yield (1095.66 kg da^-1^) was obtained from the I_30_ treatment. In terms of the genotypes, the highest yield (1430 kg ha^-1^) was obtained in the ADA-523 genotype and the lowest yield (959 kg ha^-1^) was obtained in the Kompozit Arifiye genotype.

Some researchers reported the yields of fully irrigated non-chemical treated plots to be between 10955.0–3690.0 kg ha^-1^ [5]. A different study reported the yields to be between 2650–10524.0 kg ha^-1^ [32]. In another study, the yields were reported to be between 3020.0–11020.0 kg ha^-1^ [33] and the yields of sensitive hybrids to be between 386.0–2683.0 kg ha^-1^ and the yields of resistant hybrids to be between 6803.0–9984.0 kg ha^-1^ [30].

The findings of the present study are different from earlier studies due to differences in environmental factors, climate, irrigation programs, corn genotypes, cultural practices. With regard to the European corn borer population, main plot (genotype) x sub-plot (irrigation) interactions were found to be significant (P<0.01). European corn borer populations varied between 2.7–6.82 pests plant^-1^ with the largest population in the I_100_ x Kompozit Arifiye interaction (6.82 pest plant^-1^) and the lowest population in the I_30_ x ADA-523 interaction (2.7 pest plant^-1^). While the Kompozit Arifiye genotype was placed in Group A, the ADA-523 genotype was placed in Group H. According to the variance analyses on the European corn borer populations, irrigation treatments and genotypes were also found to be significant (P<0.01). The largest European corn borer population (5.75 number plant^-1^) was observed in the I_100_ treatment and the lowest population (3.38 number plant^-1^) was observed in the I_30_ treatment. In terms of the genotypes, the largest population (5.38 number plant^-1^) was observed in the Kompozit Arifiye genotype and the lowest population (3.64 number plant^-1^) was observed in the ADA-523 genotype.

Some researchers reported the number of live larva + pupa in the 33P67 Bt (transgenic) genotype in the fully irrigated and untreated plots as 0.037 pest plant^-1^ and reported the value for isogenic (non-transgenic) 33P66 genotype as 2.220 pest plant^-1^. These values were partially similar to the values of the present study [5].

With regard to tunnel lengths, main plot (genotype) x sub-plot (irrigation) interactions were found to be significant (P<0.01). Tunnel lengths varied between 29.36–7.73 cm with the greatest value in the I_100_ x Kompozit Arifiye interaction and the lowest value in the I_30_ x ADA-523 interaction. While the Kompozit Arifiye genotype was placed in Group A, the 31D24 genotype was placed in Group K. According to the variance analyses on tunnel lengths, irrigation treatments and genotypes were found to be significant. The greatest tunnel length (18.46 cm) was observed in the I_100_ treatment and the shortest tunnel length (8.99 cm) was observed in the I_30_ treatment. Considering the tunnel lengths of the genotypes, the greatest value (21.67 cm) was observed in the Kompozit Arifiye genotype and the lowest value (9.79 cm) was observed in the ADA-523 genotype.

Researchers reported the tunnel lengths in fully irrigated untreated plots to be between 0.2–56.9 cm plant^-1^ [34]. These values were different from the values of the present study due to differences in environmental factors, climates, cultural practices, irrigation programs, genotypes, inoculations and biotic stress factors.

With regard to the number of holes 100 internodes^-1^ values, main plot (genotype) x sub-plot (irrigation) interactions were found to be significant (P<0.01). The number of holes 100 internodes^-1^ values varied between 6.88–13.70 with the greatest value in the I_100_ x ADA-523 interaction (13.70) and the lowest value in the I_30_ x Kompozit Arifiye interaction (6.88). While the ADA-523 genotype was placed in Group A, the Kompozit Arifiye genotype was placed in Group J. According to the variance analyses, irrigation treatments and genotypes were also found to be significant (P<0.01). The greatest number of holes 100 internodes^-1^ value (11.61) was observed in the I_100_ treatment and the lowest value (7.48) was observed in the I_30_ treatment. In terms of the number of holes 100 internodes^-1^ values of the genotypes, the greatest value (10.85) was observed in the ADA-523 and the lowest value (8.89) was observed in the Kompozit Arifiye genotype.

Researchers reported number of holes 100 internodes^-1^ value as 0.130 for 33P67 Bt (transgenic) genotype, 8.350 for 33P66 genotype and 8.768 for TTM 815 genotype [5]. Other researchers reported the least number of holes 100 internodes^-1^ value of fully irrigated untreated plots as 6.1 for the 9B x 24A genotype and the greatest as 80.1 for the 7C2 x 9A genotype [32]. Some researchers carried out a study on the effects of different irrigation treatments (I_0_, I_20_, I_40_, I_60_, I_80_, I_100_) on the European corn borer and Mediterranean corn borer in second crop corn under the conditions observed in Çukurova and reported number of holes 100 internodes^-1^ value of the ADA 9516 genotype as 13.50 in the I_0_ treatment and 27.167 in the I_100_ treatment [10, 34]. These findings are partially different from the findings of the present study due to differences in irrigation programs, corn genotypes, cultural practices, climate conditions and resistance of the genotypes to biotic stress.

The greatest values in the number of live larva + pupa, number of holes 100 internodes^-1^, tunnel length were obtained from the I_100_ treatment and the lowest values were obtained in the I_30_ treatment. The European corn borer population also decreased in the I_30_ treatment compared to the I_100_ treatment. The European corn borer preferred fully irrigated plants.

Correlations of the irrigations with yield, plant height, chlorophyll, plant water consumptions, water use efficiency were also investigated in this study and significant correlations were observed (p<0.01 and p<0.05) (Table 5).

**Table 5:** The correlation coefficients between irrigation (I_100_, I_50_, I_30_,) and other parameters.

	Yield (kg ha^-1^)	Plant height (m)	Chlorophyll (spad)	ETa (mm)	WUE (kg ha mm ^-1)^
Yield (kg da^-1^)					
Plant height (m)	0,13ns				
Klorofil	0,33ns	0,43*			
ETa	0,41*	0,54*	0,91**		
WUE	0,28ns	-0,48*	-0,70**	-0,74**	

* and **, significant at P≤0.05 and P≤ 0.01 level, respectively; ns, not significant; NOH/100, Number of holes /100 internodes; TL/P, Tunnel length/plant; TNOAL+PP, The number of alive larva+pupa in the plant; ETa, Plant water consumptions; WUE, Water use efficiency

The correlations coefficients (r) for the experimental year (2015) are provided in Table 5. Significant correlations were observed between some traits (p<0.01 and p<0.05). Highly significant correlations were observed between plant water consumptions and yield (r = 0.41*), plant water consumptions and plant height (r = 0.54**), plant water consumptions and chlorophyll (r = 0.91**), plant water consumptions and water use efficiency (r = -0.74**).

## Conclusions

The present study was conducted to investigate the effects of different irrigation treatments on the European corn borer populations of four corn genotypes. This study also aimed to determine the biotic stress screening parameters in the identification of the genotypes that are resistant to biotic stress. For the determination of the screening parameters, yield, tunnel length, live larva + pupa at different irrigation treatments (I_100_, I_50_ and I_30_) were used. The European corn borer preferred the I_100_ treatment the most and the I_30_ treatment the least. However, the pest did not prefer each genotype at the same level even in the I_100_ treatment. The genotype sensitive to European corn borer (European corn borer) had higher fatty acid contents and types than the genotype resistant to the European corn borer (ADA-523). In this case, the sensitive genotypes synthesized higher quantities of fatty acids than the resistant genotypes under water deficit and biotic stress. It was also determined that the genotypes with the greatest number of holes had high yield levels and the genotypes with the greatest tunnel length, high number of live larva + pupa and fatty acid contents had low yield levels. Significant correlations were observed between all investigated traits (P < 0.01 / P < 0.05).

In this study, increasing yield, number of holes 100 internodes^-^, tunnel length and live larva + pupa values were observed with increasing amount of irrigation water. Therefore, the genotypes with similar performance under both water-stressed and non-stressed conditions could be identified as resistant to the European corn borer. On the other hand, rather than the number of holes 100 internodes^-1^, tunnel length could yield more accurate outcomes in European corn borer resistance studies. In this study, the genotype ADA-523 with low tunnel lengths was identified as moderately resistant and the Kompozit Arifiye genotype with high tunnel lengths was identified as sensitive to biotic stress. Several stress screening parameters of the present study can be used in the identification of genotype resistances to biotic stressors. The present findings can further be proved with field experiments in future studies.
